# Advances in monolingual and crosslingual automatic disability annotation in Spanish

**DOI:** 10.1186/s12859-023-05372-3

**Published:** 2023-06-26

**Authors:** Iakes Goenaga, Edgar Andres, Koldo Gojenola, Aitziber Atutxa

**Affiliations:** 1 HiTZ: Basque Center for Language Technology, University of the Basque Country UPV/EHU, Donostia, Spain; 2 HiTZ: Basque Center for Language Technology, University of the Basque Country UPV/EHU, Bilbao, Spain

**Keywords:** Artificial intelligence, Neural networks, Named entity recognition, Disability annotation, Embeddings, Crosslingual learning

## Abstract

**Background:**

Unlike diseases, automatic recognition of disabilities has not received the same attention in the area of medical NLP. Progress in this direction is hampered by obstacles like the lack of annotated corpus. Neural architectures learn to translate sequences from spontaneous representations into their corresponding standard representations given a set of samples. The aim of this paper is to present the last advances in monolingual (Spanish) and crosslingual (from English to Spanish and vice versa) automatic disability annotation. The task consists of identifying disability mentions in medical texts written in Spanish within a collection of abstracts from journal papers related to the biomedical domain.

**Results:**

In order to carry out the task, we have combined deep learning models that use different embedding granularities for sequence to sequence tagging with a simple acronym and abbreviation detection module to boost the coverage.

**Conclusions:**

Our monolingual experiments demonstrate that a good combination of different word embedding representations provide better results than single representations, significantly outperforming the state of the art in disability annotation in Spanish. Additionally, we have experimented crosslingual transfer (zero-shot) for disability annotation between English and Spanish with interesting results that might help overcoming the data scarcity bottleneck, specially significant for the disabilities.

## Introduction

The International Classification of Functioning, Disability and Health (ICF) defines disability as a term which groups together a highly heterogeneous set of impairments, activity limitations and participation restrictions. People with disabilities experience increased vulnerability to secondary conditions, comorbid conditions, and higher rates of premature death, among other things due to the fact that some disabilities also cause physical and/or mental illness [1, 2].

According to the World Health Organization (WHO), 15% of the world’s population suffer some kind of disability. WHO also claims that *lack of information or data collection and analysis on disability, all contribute to health inequities faced by this group*, and *they are often left out of public health interventions* [3]. Additionally, in ontologies like UMLS disabilities do not belong to any specific semantic type, they are wide spread across different types; some belong to the *Findings*, some to the *Diseases or Syndromes* and some others to the *Mental or Behavioral Dysfunctions*. Although having an intersection with the aforementioned semantic types, they show differential characteristics like the use of less formal language, longer entities and negative polarity terms like for example *loss*, *dysfunction*, or *alteration*. These facts show the relevance and the challenges disability identification poses, requiring specific attention and research.

Medical text processing has boomed since the big increase in the availability of textual information in the form of scientific literature or Electronic Health Records (EHR). Together with the wealth of available textual information, Machine Learning and Deep Learning approaches have provided new representations and algorithms that have revolutionized the fields of Artificial Intelligence and Natural Language Processing, giving amazing improvements on the state of the art. Lately, several *word and text representation models* such as word-based, subword-based, character-based, or cross-lingual *embeddings* have emerged, together with corresponding algorithms like Seq2Seq [4] or Transformer models [5].

The recognition of Medical Named Entities (MER) is one of the basic yet crucial steps for the success of any higher level automatic tool. The goal of Named Entity Recognition (NER) is to automatically identify relevant entities in written texts, labelling each token with an entity tag. In the clinical domain, the typical entities correspond to symptoms, diseases, body parts, and drugs. The majority of the literature focuses on performing MER in English [6]. However, in recent years there has been an increasing interest in the processing of other languages (see [7] for a review of clinical NLP in languages other than English). For example, there have been recent works on the processing of Spanish, Swedish or Chinese [8–13]. Working on disabilities and especially in languages other than English is a challenging problem due to data scarcity. To our knowledge, the DIANN task [14] is the only evaluation task exclusively devoted to the automatic recognition of disability mentions. The task was divided in two sub-tasks, corresponding to the detection of disabilities in English and Spanish in a Biomedical corpus.

**Fig. 1:**
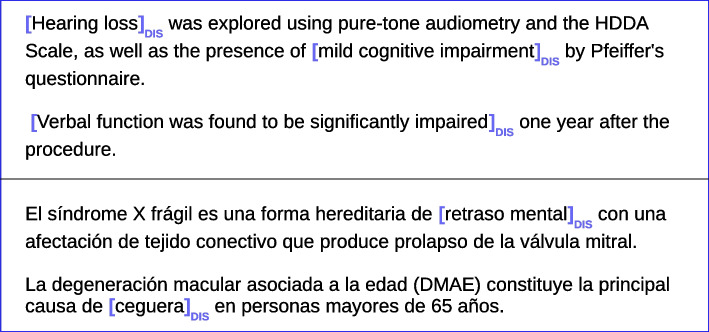
Examples of annotated disabilities (upper part in English and lower part in Spanish)

In this work, we present a set of experiments on the detection of disability mentions in Spanish (see Fig. 1). We will experiment with different approaches, thoroughly evaluating the contribution of different Deep Learning approaches and study the strengths and weaknesses of each option. Specifically, we will test the construction of textual representations like word embeddings, character-, segment- or word-based, which can be a key factor. We will also experiment with different Deep Learning algorithms, including the Transformer architecture and multilingual and cross-lingual approaches, going beyond monolingual systems. This article aims to make a new proposal based on the analysis of the distinctive features to draw conclusions about the most influential ones and their combination in effective ways.

The paper is organized as follows: the next section will examine relevant related work. In subsection *Resources* we briefly describe the corpora used for training and evaluation, including other additional textual data used. Afterwards, subsection *Techniques* analyzes and compares the different techniques and algorithms. To conclude, we present the main results and discuss the main outcomes involved.

## Related work

The CoNLL 2003 shared task [15] was a milestone concerning general purpose NER that led the way to current systems. Since then, several annotated corpora have been developed in different biomedical domains, specially for English. The entities involved depend on each task, and correspond to elements such as gene names, proteins, drugs, procedures and diseases. Regarding other languages, several annotated corpora have been used, as the IxaMed-GS corpus [13], conformed by Electronic Health Records (EHR) written in Spanish annotated with drugs and diseases. In addition to all these corpora and tasks, initiatives focused on specific and less studied types of medical entities, such as the corpus used in the DIANN shared task [14] have also emerged.

Looking at the approaches employed for NER, machine learning gave a first significant boost to the task, with powerful algorithms such as support vector machines (SVM) [16], conditional random fields (CRF) [17] or the Perceptron algorithm [18]. Examples of their application to medical NER are [19, 20] for English, [11] for Chinese and [10] for Swedish and Spanish.

In the last years, machine learning techniques have experimented a revolution with neural networks and deep learning [21, 22]. These algorithms drastically reduce the need of feature engineering, as they are able to directly learn the critical features from numerical representations of the data. There are different variants of neural network algorithms, such as recurrent neural networks (RNN) [23], long short-term memories (LSTM) [24], convolutional neural networks (CNN) [25] or Transformer Architectures [26]. A distinctive feature of neural approaches is the use of textual *embeddings* [27], which are vectorial representations that are learned in an unsupervised manner using huge amounts of unlabeled text as input. These representations provide distributional information about words and they encode relevant linguistic and semantic information. In this way, words that have the same meaning share a similar representation, and using simple operations like the cosine distance between two vectors can help to group similar concepts together. This can significantly improve the generalization ability of models learned on limited amounts of data, naturally capturing word meanings. Although the initial algorithm was originally devised for words, the use of vector operations also allows to obtain vectorial representations of characters, word pieces (also called subwords), multiword terms, sentences or even whole documents. Word2vec [27], Stanford GLOVE [28], and Facebook FASTTEXT [29] are the best known algorithms for generating word embeddings. Several works have successfully made use of these pre-trained embeddings as input to improve the performance on different tasks like general or medical NER [8, 9, 11, 30, 31]. When using pre-trained embeddings, they can be generated from out-of-domain corpora, or also from domain related corpora, in our case general medical corpora (journals or scientific abstracts), or corpora extracted from electronic health records (EHR). It is still an unresolved question to decide whether better embeddings can be obtained when training using general domain huge amounts of text or smaller in-domain corpora, which could in principle be nearer in word meaning and usage. Many times, especially in the case of clinical data, in-domain corpora is harder to obtain or simply unavailable. Current state-of-the-art methods have made use of distinct embedding types:

- *Classic* word embeddings, like GLOVE or FASTTEXT. These works [27, 28] calculate pre-trained embeddings over very large corpora trying to capture latent syntactic and semantic similarities. They have been very effective in multiple tasks.
- *Character-level* embeddings. Although most works on NLP and neural networks have taken the word as the basic processing unit, character-based information is attractive because (1) character contexts are less sparse than word contexts, and (2) characters can capture details that word-based models can not, as prefixes and suffixes that are helpful to correctly identify out of vocabulary (OOV) or misspelled words [30].
- *Subwords*. Using individual words as the basic unit discards meaningful semantic structure between words that share substructures. For this reason, apart from character-based models, byte pair encoding (BPE), a compression algorithm, has been used in several applications, like machine translation and text processing [32, 33]. Technical domains such as scientific and medical literature compose words from subword structures such as prefixes, suffixes, root-words as well as compound words, cognates and loan words. For example, *neurofibromatosis*, a complex term that could be otherwise classified as an unknown OOV word, can be given a meaning looking at its affixes *neuro-* and *-osis*, and classify it as a disease related to neurons, if embeddings were calculated taking those subwords as unit.
- *Multilingual* embeddings. They provide a way to transfer and share knowledge across different languages, thus porting information from languages with more resources to underresourced ones [34]. There are two main approaches:
  - Simultaneous training of a single language model (LM) using multiple languages, allowing to profit of bigger training corpora. This way, cross-lingual learning can be applied, where the cross-lingual model is fine-tuned in one of the languages and then used in zero-shot scenarios where there is no training data for the other languages.
  - Training each language’s embeddings independently and a posterior alignment in a common space by means of linear transformations and bilingual dictionaries. The main idea is to learn a mapping from the source to the target space using an iterative alignment method, giving as a result a multilingual representation

Regarding the types of software architectures used, we can distinguish the following ones:

- *Sequential* architectures [35]. These systems presented a first breakthrough [30] on the NER task by means of neural networks applied to sequential tagging, using a bidirectional BiLSTM architecture followed by a Conditional Random field layer (CRF) that models joint tag dependencies, surpassing the previous state of the art by a significant margin. They take pre-trained word embeddings as additional input for training, and character embeddings internally for the detection of prefixes and suffixes. We can distinguish two main types regarding the context they use: (a) Static embeddings. This was the first type of models [30, 31] that made use of pre-trained word embeddings. Although the results improved the best current systems, one disadvantage is that using this approach each word form is assigned a single vector containing its representation independent from its context. (b) Contextualized or *dynamic* embeddings [36–38] capture semantics in context to address the polysemous and context-dependent nature of words. These dynamic embeddings are calculated taking the context into account, that is, the same word can receive different embeddings depending on its context.
- *Transformer-based* architectures [26] use the attention mechanism to account for the context of each word. In Recurrent Neural Networks or LSTMs, the importance of the past elements can vanish with distance. Using transformers, instead of sequentially applying the same network, the idea is to connect the current token to all the elements, preceding and posterior, where each element has a positional embedding concatenated to it. The aim is to incorporate the context in the processing of the current word, by a mechanism that weights the relevance of each context word with respect to the current one. This technique has produced state-of-the-art models while at the same time decreasing training time due to an easier parallelization.

The DIANN shared task [14] was dedicated to the detection of disability mentions in biomedical research texts in English and Spanish, with the objective of evaluating the performance of various named entity recognition systems in two different languages. In the first position, [39] presented a neural network-based architecture system consisting of a bidirectional long short term memory network (BiLSTM) and a conditional random field (CRF), using static word embeddings for both languages combined with a rule-based acronyms and abbreviation module for the detection of disability-related acronyms and abbreviations, obtaining an F-measure of 0.82 and 0.78 for English and Spanish, respectively. [14] uses a long short-term memory architecture for disabilities, improving the state of the art, with an F-measure of 0.83 and 0.81 for English and Spanish. More recently, [40] present experiments on this corpus incorporating negation-based transfer learning to disability annotation. Although the use of negation information considerably improves their baseline system, it is still below the state of the art (they reach an F-measure of 76.9 and 76.5 for English and Spanish).

**Table 1:** General data on the DIANN annotated corpus of disabilities and rare diseases

	Documents		Tokens		Disabilities	
	Train	Test	Train	Test	Train	Test
Spanish	400	100	70,919	18,406	1413	243
English	400	100	78,381	20,567	1326	229

## Materials and methods

In this section we will explore all the corpus and tools we have used in order to carry out the experiments. The first subsection describes the data, which includes annotated data and raw text. The next two subsections will present, respectively, the Deep Learning and Rule-based approaches that have been implemented, concluding with a description of the main experimental settings in the last subsection.

### Resources

In this subsection we will first present the DIANN annotated corpus of disabilities, and the unannotated additional texts that we have used in our experiments for Spanish and English in subsection *Embeddings*.

#### The DIANN annotated corpus

The DIANN corpus [14] is a gold standard corpus annotated with disabilities. The corpus includes 500 abstracts from scientific papers corresponding to the biomedical domain between the years 2017 and 2018, related to rare diseases. The document compilation was restricted to documents with the abstract in both English and Spanish containing at least a disability in both languages.

Disabilities are commonly expressed either with a specific word, such as *blindness*, or as the limitation or absence of a human function, such as *lack of vision*. The corpus^1^ is publicly accessible and it will allow to train machine and deep learning systems, thus extracting new information about the relations between rare diseases and disabilities. Table 1 presents the main characteristics of the corpus.

Some disabilities are mentioned more than 50 times whereas others are mentioned only once, with an average of 1.8 mentions for each disease. From them, 72% are expressed as the impairment of a human function, while 23% are stated using some disability term. In 5% of the cases, the disability corresponds to an acronym. The most frequently mentioned disability is *ataxia*, related to motor skills, followed by *deafness*, *dementia* (related to problems in cognitive functions), *autism* and *blindness*. The most frequent physical impairment functions are associated to hearing, sight and motor skills, affection of cognitive capacities and related to development.

Although the annotated disabilities have a common intersection with clinical categories like UMLS diseases or disorders, there are also important differences. For example, disabilities are presented in longer sequences (19.79 characters and 2.29 words on average per disability) compared to diseases (12.39 characters and 1.43 words per disease in the Spanish IxaMed-GS [13] corpus). We did preliminary experiments using a state of the art clinical NER system for Spanish [41], and found that it was able to correctly detect only 31% of the disabilities. This can be explained by the less specialized language used for disabilities compared to current medical NER diseases. For example, disabilities like *mental disorders*, *problems in working memory* or *capacity limitations in phonological working memory* could not be detected by the standard clinical NER system, which otherwise has an f1-score of 90% for diseases.

#### Embeddings

Deep learning techniques usually require huge amounts of data. Although manually annotated data give the best results, it is very expensive and time consuming. For that reason, the idea of acquiring useful information in an unsupervised manner through *embeddings* is very attractive, and efficient and effective methods have been developed. This way, a system can have information on the fact that, for example, *infarct* and *stroke* are similar terms, even when the latter did not appear in the annotated corpus.

With this objective, we made use of several other corpora for adding unsupervised knowledge to the system, either directly processing textual corpora to obtain different embeddings, or indirectly through the use of pre-calculated embeddings. This allows to measure the impact of using general available resources or domain specific ones.

For the latter case, we made use of a EHR corpus (the Spanish EHR corpus henceforth) that comprises 300,000 unnanotated EHRs collected over 4 years during the period 2012–2016 at the regional Hospitals from the Basque Health System, with approximately 200 million tokens. The corpus consists of deidentified patient records subject to a confidentiality agreement. The EHRs follow the standard SOAP notes method (Subjective, Objective, Assessment, Plan) and they are semistructured. In order to experiment with a varied number of possibilities, we have tested the following types of embeddings for the monolingual setting (training and test on the same language, Spanish in our case):

- FASTTEXT pre-trained Embeddings [29], trained on the Spanish Wikipedia (797 M tokens) and CommonCrawl (72,000 M tokens).
- Wikipedia2Vec pre-trained embeddings [42], of words and entities from Wikipedia. This tool enables users to learn the embeddings giving a Wikipedia dump file as argument.
- SkipNGram word embeddings [43] trained from the Spanish EHR corpus. In order to better model the language contained in our EHRs, we trained our own LM. Although this corpus is smaller than the previous ones, it has the advantage of containing in-domain text, which can be helpful for many tasks.
- Flair contextualized character embeddings trained from the Spanish EHR corpus [38].
- Transformer-based LM [44]. BETO is a BERT [45] model trained on a big general Spanish corpus (Wikipedia and news, among others), similar to a BERT-Base in size and trained using the Whole Word Masking technique.

For the cross-lingual setting (training on one language and evaluating on the other one) we chose the following:

- MUSE [46] is a library designed with the goal of providing state-of-the-art multilingual static word embeddings (FASTTEXT embeddings) aligned in a common space by means of large-scale high-quality bilingual dictionaries.
- Meta-embeddings [47] integrate multiple word embeddings created from complementary sources such as text or knowledge bases, projecting word vectors to a common semantic space using linear transformations and averaging. They combine, for English, Word2Vec embeddings from Google News (100 billion words), GloVe and FastText from Common Crawl (600 billion words), while for Spanish they use the vectors trained on the Spanish Billion Word Corpus (1.4 billion words).
- Multilingual BERT (mBERT) provides contextual embedding representations for 104 languages, which have been applied to many multilingual or cross-lingual tasks [45].
- XLM-RoBERTa (XLM-R) [48] is a transformer-based language model, pre-trained on general domain texts in 100 languages, based on subword embeddings.

**Fig. 2:**
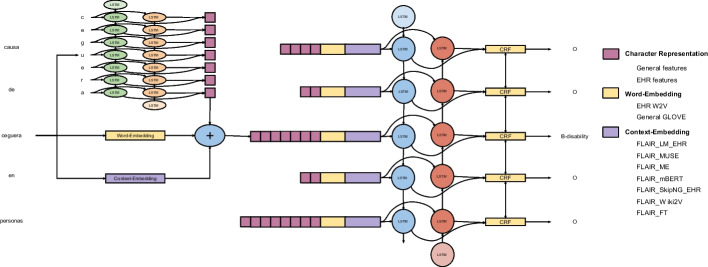
Main architecture of the system

### Deep learning: monolingual and multilingual approaches

Based on the resources described in the previous subsection, we have experimented with the different options presented using state-of-the-art neural architectures. On the one hand, for the evaluation of the contribution of different embedding types in the monolingual setting, we have chosen the Flair architecture, built upon contextual character embeddings. For the crosslingual experiments, we have tested the multilingual extensions to *Flair* and the transformer-based XLM-R architecture (see next subsection). Figure 2 presents the main architecture we have used, including different types of embeddings, character, word and contextual (see left side of the figure), a bidirectional LSTM layer (middle) and a final CRF layer that will produce the final output.

#### Contextualized string embeddings

Akbik et al. [38] propose a contextualized character-level word embedding model, that tries to combine the best attributes of different embedding types. Their framework allows the testing of different NLP models, such as NER, part-of-speech tagging (PoS), and classification on a given text. The system’s most distinguishing features are:

- The texts are modelled as sequences of *characters* instead of words using a standard sequential BiLSTM-CRF model. This radical approach will allow to better handle OOV and misspelled words as well as substructures such as prefixes and suffixes. Even when the system is based on character embeddings, it is able to generate an embedding for any string of characters [37]. For example, a word can be modelled as the concatenation of the output hidden state after the last character in the word in the forward LM and the hidden state of the first character in the word in the backward LM.
- The ability to pre-train on large unlabeled corpora. This way, we can either make use of pre-trained LM embeddings calculated over huge volumes of text (such as BERT embeddings [45], ELMo embeddings [36], FASTEXT embeddings [29], or Flair embeddings [37]) or otherwise generate a new LM based on each user’s own unannotated data. These general pre-trained LMs, also known as stacked embeddings, can be fine-tuned to specific tasks by a second round of training on the final objective with successful results.
- As the embeddings are contextual, they capture word meaning in context, producing different embeddings for polysemous words depending on their usage.

#### Multilingual transformers

XLM-RoBERTa (XLM-R) [48] makes use of a transformer-based multilingual masked language model, pre-trained on text in 100 languages, that obtains state-of-the-art performance on several NLP tasks, including sequence labeling. Contrary to the alternative used in Flair, the LM used by this system has been simultaneously trained on text from all the languages.

**Table 2:** Overview of the different approaches used for automatic disability annotation, tested on Spanish data

Main architecture	System	Stacked external embeddings	Level of granularity	Train/dev
*Monolingual approaches*				
BiLSTM-CRF	2018 Shared task Best system [49]	W2V static word + character features (EHR)	Static word	DIANN Spa
	Best published Result [14]	GLOVE static word + character features (general texts)		
	$FLAIR_{FT}$ [37]	FASTTEXT Static word & subword (general texts)	Contextual character	
	$FLAIR_{Wiki2V}$ [37]	Wikipedia2Vec Static word		
	$FLAIR_{SkipNG\_EHR}$ [43]	SkipNG Static word (EHR)		
	$FLAIR_{LM\_EHR}$ [37]	FLAIR contextual Character (EHR)		
Transformer	*BETO* [44]	Spanish contextual subword	Spanish contextual subword	
*Crosslingual approaches*				
BiLSTM-CRF	$FLAIR_{MUSE}$ [46]	Bilingual static subword	Contextual character	DIANN Spa/Eng
	$FLAIR_{ME}$ [47]	Static word		
	$FLAIR_{mBERT}$ [45]	Contextual subword		
Transformer	$XLM-R$ [48]	Multilingual contextual subword	Multilingual contextual subword	

The upper table presents the experiments with monolingual approaches (training with Spanish data) and the lower table the ones using crosslingual approaches (from English to Spanish and vice versa)

The unit used for processing is the subword (also called *word piece)*, which allows to decompose a word into smaller components, ideal for generalizations, OOVs, misspellings and crosslingual processing. Different languages can share subvocabularies, either literally or by means of local transformations, and this is more usual for specialized subdomains such as medicine (Table 2).

### Acronym and abbreviation detection module

In order to detect some disabilities represented by acronyms that deep learning techniques are not able to identify, we have created a rule-based acronym and abbreviation detection module. This module is responsible of detecting the acronyms of disabilities that are close to the disabilities (maximum one word distance) identified by the neural network. To be detected as an acronym, the acronym must be in parentheses and have only capital letters (more than one capital letter). Once the acronyms are detected the module labels them as disabilities in the entire text. Table 3 shows an example of the application of the rules. In the first case, the deep learning methods fail to capture the acronym (CP). In the second case (low part of the table) the addition of rules allows to detect two instances of the CP disability.

**Table 3:** Example of disability identification using deep learning (above) and using the acronym and abbreviation detection module (below). Identified disabilities are shown in bold

**Disability Identification (Deep Learning)**
There are many instruments designed to evaluate motor function in children with **cerebral palsy** (CP)... motor function over time in children with CP
**Disability Identification (Deep Learning + Rules)**
There are many instruments designed to evaluate motor function in children with **cerebral palsy (CP)**... motor function over time in children with **CP**

### Experimental settings

Table 2 presents the different types of systems that will be compared in this paper. On the monolingual part (upper side of Table 2) we describe a set of experiments that use the Spanish DIANN corpus for training, development and test. The first two systems correspond to the best published results until the moment, which are both based on using static word embeddings and a BiLSTM-CRF architecture for training, representing the current state of the art. The next systems use the Flair framework of contextualized character embeddings taking different external embedding sources: FASTTEXT ($FLAIR_{FT}$), Wikipedia2Vec ($FLAIR_{Wiki2V}$), FLAIR’s Wikipedia-based pre-trained embeddings ($FLAIR_{LM\_Wiki}$), SkipNGram static word embeddings pre-trained on our own Spanish EHR corpus ($FLAIR_{SkipNG\_EHR}$), and FLAIR contextual character EHR embeddings ($FLAIR_{LM\_EHR}$). For the sake of comparison with a transformer model, we have also added BETO [44].

As the best performing systems [14, 49] have used a combination of a Deep Learning base system and an acronym and abbreviation module inspired in ours to improve the results, in the next section (see *Results*) we will also present the results with and without this module, for the sake of comparison. Additionally, we have also tested the usage of several combinations of external (or pre-trained) embeddings to train new sequence labeling and text classification models, thus trying to incorporate complementary types of knowledge into the system. We experimented with the two and three best performing embedding types (B2/B3 for best two/three embedding types, presented in the lower part of Table 4).

The lower part of Table 2 presents the crosslingual experiments, where a system takes as input a multilingual representation that includes both English and Spanish mapped into a single embedding space together with the English DIANN annotated corpus and applied to the Spanish DIANN test set. These experiments can show to what degree a system can be derived to a target language (Spanish) with no annotated data in that language, using a source language (English) with more annotated resources.

In the results we will provide the average and standard deviation of several evaluation rounds with different initialization seeds, to give an estimation about the variability that can be found when replicating the experiments, as pointed out in [50]. A cumulative of 133 h of computation was performed on hardware of type Titan V (TDP of 250W) with 12 GB of RAM. Total emissions are estimated to be 14.36 kgCO$_2$ of which 0 percents were directly offset. Estimations were conducted using the MachineLearning Impact calculator.^2^

**Table 4:** Monolingual experiments

System	Precision		Recall		F-measure	
	A−	A+	A−	A+	A−	A+ (stdev)
2018 Shared task best system [49]		75.00		81.00	71.46	78.60
Best published result [14]	79.00	83.00	69.00	79.00	74.00	81.00
*BETO*	77.73	78.41	66.57	74.22	71.64	75.98 (±2.15)
$FLAIR_{SkipNG\_EHR}$	83.23	83.05	73.07	80.64	77.82	81.82 (±1.51)
$FLAIR_{FT}$	84.90	83.67	71.18	81.22	77.43	82.43 (±1.28)
$FLAIR_{Wiki2V}$	85.63	84.27	76.27	80.78	80.67	82.64 (±0.37)
$FLAIR_{LM\_EHR}$	84.53	85.66	72.20	83.26	77.87	84.43 (±0.93)
*Combined approaches*						
$FLAIR_{B2}$ ($FLAIR_{LM\_EHR}$ + $FLAIR_{Wiki2V}$)	87.61	**87.68**	76.13	**85.88**	81.47	**86.77** (±0.50)
$FLAIR_{B3}$ ($FLAIR_{LM\_EHR}$ + $FLAIR_{Wiki2V}$ + $FLAIR_{FT}$)	87.67	87.66	73.51	82.83	79.96	85.16 (±1.59)

Results of the different approaches used for automatic disability annotation in Spanish (the best results are presented in bold). A−: without Acronym and abbreviation module. A+: with the Acronym and abbreviation module. The upper part of the table shows the results using a single source of pre-trained embeddings, while the lower part presents the combinations of the best two (B2) and three (B3) embedding types

## Results

The upper part of Table 4 presents the results for the monolingual approaches using different pre-trained embeddings and training and test being performed on the Spanish DIANN corpus. The first two lines give the best reported results in the literature [14, 49]. The best system at the 2018 DIANN shared task used a BiLSTM-CRF with general domain static word embeddings and obtained an F-measure of 78.60 [49], while [14] improved this basic architecture by adding character embeddings and a casing embedding vector, reaching an F-measure of 81.00. The table shows how using a transformer base general domain Language Model (BETO) do not surpass even the shared task best results. Using pre-trained static word embeddings based on in-domain EHRs ($FLAIR_{SkipNG\_EHR}$) the results are better than the best reported systems. The FASTTEXT ($FLAIR_{FT}$) and Wikipedia2Vec ($FLAIR_{Wiki2V}$) embeddings give a slight increase in the results. In the last line, we see that the addition of pre-trained contextualized character embeddings based on EHRs ($FLAIR_{LM\_EHR}$) gives a final significant improvement (F-measure of 84.43) over the previous results, with a noticeable increase in all the measures.

The lower part of Table 4 presents the combination of the two and three best ($FLAIR_{B2}$ and $FLAIR_{B3}$) embedding types, which give an additional boost in both precision and recall and obtain the best result (86.77 F-measure). We must note that the combination does not require the independent training of different systems, and instead a single training phase providing the different embedding types is necessary.

Table 5 shows the results for the crosslingual approaches in a zero-shot setting where there is no annotated data in the target language (Spanish or English) and the system relies on multilingual aligned embeddings and training on the source language. The $FLAIR_{ME}$ system gets the best balanced compromise between precision and recall, with an F-measure of 46.31 and 52.34 for Spanish and English, respectively. The multilingual BERT and XML-R systems, however, are far from the other two systems.

**Table 5:** Crosslingual experiments (zero shot)

	System	Precision		Recall		F-measure	
		A−	A+	A−	A+	A−	A+ (stdev)
	$FLAIR_{MUSE}$	70.51	76.11	23.72	27.94	35.43	42.58 (±4.40)
Train ENG	$FLAIR_{ME}$	54.65	61.24	28.67	37.70	37.31	46.31 (±2.31)
Test SPA	$FLAIR_{mBERT}$	35.04	38.85	10.04	12.66	13.98	17.12 (±9.67)
	$XLM-R$	48.09	58.04	2.57	28.61	29.49	37.51 (±9.35)
	$FLAIR_{MUSE}$	57.08	59.41	23.32	35.01	32.89	43.71 (±2.87)
Train SPA	$FLAIR_{ME}$	64.71	69.95	28.40	41.84	39.45	52.34 (±1.44)
Test ENG	$FLAIR_{mBERT}$	17.53	22.70	13.03	19.76	14.49	20.45 (±13.21)
	$XLM-R$	18.93	28.26	6.16	10.49	8.71	14.28 (±4.82)

Results of the different approaches used for automatic disability annotation in Spanish trained on English data. A−: without Acronym and abbreviation module. A+: with the Acronym and abbreviation module

## Discussion

In the following, the first subsection (Analysis) will comment the main features of the results presented in Tables 4 and 5. Next, we will try to inspect the results and understand the main errors, differences and improvements obtained in different models.

### Analysis of the results

The results in Table 4 show how choosing the right representation and pre-trained embedding types has a significant effect on the results. In the upper part of the table, describing the monolingual experiments, we see that a fine tuned Spanish transformer Language Model (BETO) does not reach the performance of the systems using BILSTM-CRF character-based LM (FLAIR) for this task. This is relevant because currently many implemented systems use transformers where other architectures like BI-LSTM should not necessarily be abandoned. This goes in the line of the experiments in [51] where the authors conclude that general-purpose transformer-based models are not always necessarily better than simpler approaches. Adding domain specific static embeddings, in this case based on EHRs ($FLAIR_{SkipNG\_EHR}$), although it gives an improvement over the state of the art, the obtained score is slightly lower than that of systems pretrained on larger general corpora like $FLAIR_{FT}$ and $FLAIR_{Wiki2V}$. This seems to show that the inclusion of domain specific knowledge contributes unequally depending on the nature of the knowledge; contextualized character-based embeddings generalize better as many other authors concluded already. In-domain medical EHR embeddings ($FLAIR_{LM\_EHR}$) improves substantially (more than 3 points) the best published result (81.00), while static in-domain embeddings ($FLAIR_{SkipNG\_EHR}$) do not reach a significant improvement.

The lower part of Table 4 presents the results when several embedding types are combined, using the best two ($FLAIR_{B2}$) or three ($FLAIR_{B3}$) types of embeddings (with an score of 86.28 and 87.05, respectively). We must note that the combined systems are not the result of training different systems, but they use a single training phase taking different types of embeddings as input.

Regarding the crosslingual experiments (see Table 5) the MUSE-based system obtains the highest precision for Spanish at the cost of a lower recall. The mBERT-based and the XLM-R systems suffer from a low recall, while the Meta-embeddings-based system gives the best F-measure. Although the results are still far from being useful in any application, they present a promising avenue of research. These results show that giving good quality crosslingual embeddings trained on huge amounts of text in an unsupervised manner can be useful to port annotated knowledge from one language to another without the need of annotating the target language. It seems that the potential of models trained on one language to generalize to other languages depends on factors like language proximity, because the relatively good results obtained in our case contrast to other works [52] that showed much poorer results in Russian-English transfer on EHRs (3.07 F-score for diseases in EN $\rightarrow$ RU and 0.97 for RU $\rightarrow$ EN).

### Error analysis

We have inspected the results of the different systems trying to elucidate the varied types of information managed by each approach. Table 6 presents different instances of disabilities that are captured by each model.

The first row in the table presents an example where the weaker models are unable to correctly detect the entity, while the more powerful model ($FLAIR_{LM\_EHR}$) and the combined model can identify it. In the second and third rows we see how more powerful models in general can improve the results of the less powerful ones, although in some cases there are divergences, as in rows 4 and 5 where the $FLAIR_{Wiki2V}$ model outperforms the $FLAIR_{SkipNG\_EHR}$ one. Rows 6, 7 and 8 present several examples of entities that are detected using the models based on EHRs. Finally, the last two lines of the table present examples where the combined knowledge contained in the different individual models can be leveraged to obtain a correct analysis.

**Table 6:** Error analysis, monolingual experiments

	Entity (gold)	*FLAIR*	*FLAIR*	*FLAIR*	*FLAIR*	*FLAIR*
		FT	Wiki2V	SkipNG_EHR	LM_EHR	B2
1	Déficits en la función Ejecutiva primaria	✕	✕	✕	✓	✓
2	Alteraciones de las Funciones cognitivas	✕	✓	✓	✓	✓
3	Afectación de la memoria	✕	✓	✓	✓	✓
4	Afectación de las funciones ejecutiva y visuoespacial y de las praxias	✕	✓	✕	✓	✓ (partial match)
5	Deficiencia visual	✕	✓	✕	✓	✓
6	Retraso global del desarrollo	✕	✕	✓	✓	✓
7	Pérdida brusca de visión	✕	✕	✓	✓	✓
8	Trastornos del movimiento	✕	✕	✓	✓	✓
9	Disfunción sensoriomotora	✓	✓	✕	✕	✓
10	Patologías psiquiátricas	✓	✓	✕	✕	✓

Examples of different instances of disabilities and the result of the different models. 1. deficits in primary executive function, 2. alterations of cognitive functions, 3. memory impairment, 4. impairment of executive and visual-spatial functions and praxia, 5. visual deficiency, 6. overall developmental delay, 7. sudden loss of vision, 8. movement disorders, 9. sensorimotor dysfunction, 10. psychiatric pathologies

Table 7 presents examples correctly and incorrectly identified entities in the crosslingual setting using the best approaches of Meta-embeddings ($FLAIR_{ME}$) and MUSE ($FLAIR_{MUSE}$). Examples 1–3 show how a system trained in a different language (English) can still be useful when applied to a different language (Spanish). One of the main reasons is that using subword elements such as characters or word pieces can be specially helpful in specialized domains such as medicine, where many terms and words share prefixes, suffixes and infixes (*-neuro, sensorial, fronto-, -temporal, bilateral,...*) that help to bridge the gap between the two languages. In general, $FLAIR_{MUSE}$ gives a high precision although with a low recall, while $FLAIR_{ME}$ obtains a better balance between precision and recall. Rows 6 and 7 present examples where both systems fail, and we can see how in these examples the difference between the English and Spanish terms is bigger, which can be the cause of failure.

**Table 7:** Error analysis, crosslingual experiments (in the case of a partial matching, the overlapping span appears in bold)

	Entity (gold)	${\textbf{FLAIR}}_{{\textbf{ME}}}$	${\textbf{FLAIR}}_{{\textbf{MUSE}}}$
1	Sordera neurosensorial (*neurosensorial deafness*)	✓	✓
2	Demencia frontotemporal (*frontotemporal dementia*)	✓	✓
3	**Sordera bilateral** **neurosensorial congénita** **Y pérdida progresiva** de visión (*bilateral sensorineural* *deafness and visual* *impairment*)	✓ (partial)	✓ (partial)
4	Trastorno neuropsiquiátrico (*neuropsychiatric disorders*)	✓	✕
5	**Pérdida total** o parcial de la visión (*partial or complete* *vision loss*)	✓ (partial)	✕
6	Alteraciones del movimiento (*movement disorders*)	✕	✕
7	Trastornos psiquiátricos (*psychiatric disorders*)	✕	✕

Apart from looking at the detection of correct terms presented in Table 7, we have also examined the entities that were incorrectly detected by the $FLAIR_{ME}$ system (False Positives):

- *patologías auditivas* (auditory pathologies)
- *posibles déficits cognitivos* (possible cognitive deficits)
- *peor funcionamiento cognitivo* (worse cognitive functioning)
- *parálisis supranuclear progresiva* (progressive supranuclear palsy)
- *trastorno bipolar* (bipolar disorder)

We can see how, even when these entities do not exactly correspond to disabilities, they are instances of diseases, which can be semantically situated near disabilities, and show how the crosslingual embeddings convey the meaning associated to illnesses in some sense. In other cases, however, the system incorrectly marks some entities as disabilities when they correspond to a non-negative quality, like in potential disabilities “*posibles deficits cognitivos*” or in tests and measurements (“*Se evaluó el funcionamiento cognitivo*)”.

## Conclusion

In this work we have tested the effect of different types of embedding granularities like static word embeddings, subword embeddings and contextual character embeddings for Named Entity Recognition of disability mentions in medical texts written in Spanish. This presents a low resource scenario regarding to both the language (Spanish with respect to English) and also the subdomain (disabilities compared to diseases or medications). We have thoroughly evaluated the contribution of different Deep Learning approaches and study the strengths and weaknesses of each option. Specifically, we show that the construction of word embeddings, character-, segment- or word-based, is a key factor for the improvements.

In the monolingual setting, our system significantly outperforms the state of the art in disability annotation in Spanish, using contextual character embeddings trained on a corpus of the clinical domain (EHRs), with an F-measure of 84.43 comparing to a best reported result of 81.00. Our experiments have shown that a fine tuned Spanish transformer Language Model (BETO) is not necesarilly better than a BILSTM-CRF character-based LM (FLAIR) for this task. Although domain specific knowledge improves the results, its inclusion contributes unequally depending on the nature of the knowledge; contextualized character-based embeddings generalize better, while static in-domain embeddings are far from obtaining similar results.

We show that dynamic contextual character-based embeddings give the best performance. Additionally, we also study combinations of different embedding types forming ensembles, studying whether they convey complementary or redundant information (F-measure of 86.77). In this case more does not mean better, as the ensemble of the three best embeddings obtains worst results than the combination of just the two best ones.

We have also experimented the feasibility of crosslingual transfer (zero-shot) for disability annotation between English and Spanish, with promising results. One of the aims of this work was to explore to which extent cross-lingual knowledge might help transferring medical information across typologically distant languages to overcome data scarcity in one of the languages, Spanish in this case, showing that this can be a good starting point when there is no annotated data in the target language.

## Data Availability

The obtained material (embeddings, acronym and abbreviation module and software) will be available from the corresponding author on request.

## Footnotes

1 https://github.com/gildofabregat/DIANN-IBEREVAL-2018.

2 https://mlco2.github.io/impact/#home.
